# Determination of flumequine enantiomers and 7-hydroxyflumequine in water and sediment by chiral HPLC coupled with hybrid quadrupole-time-of-flight mass spectrometer

**DOI:** 10.1038/s41598-018-25889-5

**Published:** 2018-05-15

**Authors:** Moyong Xue, Yuchang Qin, Xu Gu, Junguo Li, Yunfeng Gao, Xiaowei Yang, Ting Yao, Zhen Zhao

**Affiliations:** 1 Feed Research Institute, Chinese Academy of Agricultural Science, Beijing, 100081 China; 2 Institute of Animal Science, Chinese Academy of Agriculture Sciences, Beijing, 100193 China; 3 Institute of Food and Nutrition Development, Ministry of Agriculture, Beijing, 10081 China; 4 Supervision Institute of Veterinary Medicine and Feed of Tianjin, Tianjin, 300402 China; 5 Beijing Institute of Feed Control, Beijing, 100012 China

## Abstract

A liquid chromatography-tandem mass spectrometric (LC-MS/MS) method for simultaneous enantiomeric analysis of flumequine and its metabolite 7-hydroxyflumequine in water and sediment had been developed based on the separation method. Sediment samples were extracted with ACN and EDTA-Mcllvaine buffer solution (40:60, v/v) then were enriched and cleaned-up by Cleanert PEP solid-phase extraction cartridges. The extract solvent, solid cartridges, mobile phase ratios, and chiral separation column were all optimized to reach high sensitivity and selectivity, good peak shape, and satisfactory resolution. The results showed that the calibration curves of flumequine enantiomers and 7-hydroxyflumequine were linear in the range of 1.0 to 200.0 µg/L with correlation coefficients of 0.9822–0.9988, the mean recoveries for both the enantiomers ranged from 69.9–84.6% with relative standard deviations (RSDs) being 13.1% or below. The limits of detection (LODs) for both flumequine enantiomers were 2.5 µg/L and 5.0 µg/kg in water and sediment samples, whereas the limits of quantification (LOQs) were 8.0 µg/L and 15.0 µg/kg, respectively. While the LODs for 7-hydroxyflumequine were 3.2 µg/L in water samples and 7.0 µg/kg in sediment samples. The proposed method will be extended for studies on the degradation kinetics and environmental behaviors and providing additional information for reliable risk assessment of these chiral antibiotics.

## Introduction

China produces antibiotics about 210,000 tons of each year, of which 48 percent are used in agriculture and animal husbandry^1^. China is the largest producer user of antibiotics in the world based on the market sales data^2–5^. Besides, China leads the world in the consumption of antibiotics in the livestock-breeding industry^6^. In the process of aquaculture, veterinary antibiotics are usually used as feed additives to improve animal growth rate and feed conversion rate^7,8^, it accounts for more than 70% of the aquatic product dosage^9^. The antibiotics which used in aquaculture cannot be completely absorbed by animals, and 70% to 80% of them enter the water body or sink into the sediment^10^, and it still have antibacterial, gene mutation activity and so on, resulting in environmental pollution and ecological damage. The heavy uses of veterinary antibiotics generated high levels of antibiotic residues in China. So far, it has been reported that antibiotic residues in the surrounding environment of animal breeding farms are detected^11,12^. Large amounts of antibiotic residues in sewage environments^13^, estuaries and coastal waters^14,15^. In the Pearl River waters of China, residual concentrations of quinolones antibiotics such as ofloxacin and norfloxacin have reached the level of mg/L^16^. In addition to the aquaculture environment, fluoroquinolone antibiotic residues have been found in other natural environments such as reservoirs, lakes and rivers in China, with concentrations ranging from 1–100 ng/L^17,18^. There are different levels of antibiotic residues in water, sediments, aquatic plants and animals of Baiyang Lake in China, the concentration level can reach up to mg/L^19^. Moreover, the concentration of oxytetracycline, tetracycline, norfloxacin and ofloxacin were detected in the sediments of the Zhujiang River were 100, 72.6, 1120 and 1560 ng/g, respectively^16^.

The accumulation of antibiotic residues in water and sediments in freshwater aquaculture areas in China, which can inhibit the decomposition ability of soil and cause harm to aquatic organisms. As well as can induce the production of resistant bacteria and can alter microbial activity and community composition in groundwater^20^. The residual antibiotics in water environment may be absorbed by the absorption and enrichment of the aquatic animals and plants^21–23^. Meanwhile, it will lead to serious environmental problems including ecological risk and human health damage^24^.

Fluoroquinolones are a well-known class of orally deliverable, bactericidal, broad spectrum antimicrobial agents that are currently used to treat serious bacterial infections^25^, which are especially active against Gram-negative bacteria and they are used both in human and animals^26^. Some fluoroquinolones available for clinical use or in development have one or two chiral centers in their chemical structure. These compounds are available either as racemates (ofloxacin, gemifloxacin, clinafloxacin), enantiomers (levofloxacin, moxifloxacin) or diastereoisomers (sparfloxacin)^27,28^.

**Figure 1:**
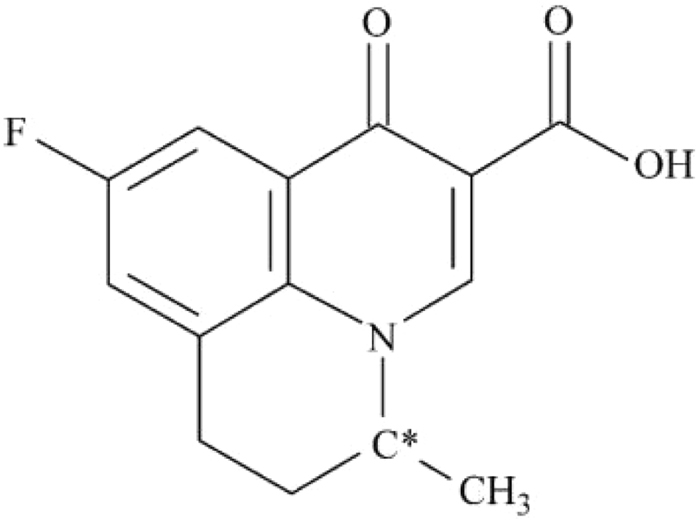
Chemical structure of flumequine (C*=chiral center).

**Figure 2:**
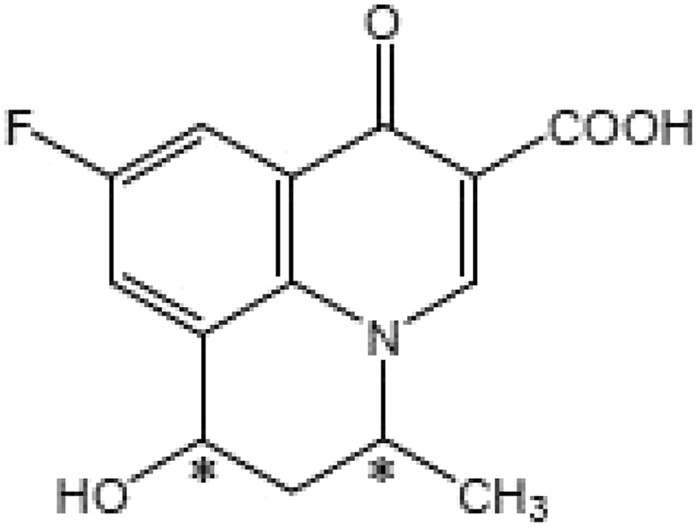
Chemical structure of 7-hydroxyflumequine (C*=chiral center).

Flumequine (9-fluoro-6, 7-dihydro-5-methyl-l-oxo-1H, 5H-benzo[ij]quinolizine-2-carboxylic acid^29^) is one of the most commonly used second-generation quinolones antibiotics, which has one chiral carbon and consists of a pair of enantiomers (Fig. 1). Its absolute configuration was confirmed with *S*-(−)-Flumequine and *R*-(+)-Flumequine^30^. The main mechanism of flumequine is to inhibit the DNA gyrase for the necessary of the cell replication, thereby blocking the replication of bacterial DNA to achieve the purpose of the sterilization^31^. Flumequine has a good effect on diseases caused by infection with Aeromonas salmonicida, Escherichia coli, which are especially active against Gram-negative bacteria and is widely used in the treatment of systemic infectious diseases in livestock and aquatic animals^32,33^. Studies have shown that high doses of flumequine can cause distortion of rat embryonic development^34^. There is no report that low doses of flumequine are harmful to animals and humans, but if people intake antibiotic residue food for long time, the potential risk can not be underestimated. Therefore, the residue of flumequine has aroused widespread concern at home and abroad^35^. 7-hydroxyflumequine is the main metabolite of flumequine, which has two chiral carbon and consist four enantiomers (Fig. 2). A survey has shown that 60–70% of the most frequently prescribed drugs and the drug candidates under development are single enantiomers^36^. For any enantiomeric drug, an enantiomeric impurity can produce different pharmacological, toxicological, metabolic, and pharmacokinetic effects within the chiral environment of a biological system^37–39^. Studies have shown that there is a significant difference in the antibacterial activity of the flumequine enantiomers^40^. Therefore, it is necessary to analyze and detect antibiotics, and further, concerned with it’s enantioselective behaviors in the environment.

In the present study, we report on the development of using the Lux 5 µm Cellulose-2 column to separate flumequine enantiomers and develop a method based on solid-phase extraction followed by high performance liquid chromatography-electrospray time of flight mass spectrometry for the determination of flumequine antibiotic. Meanwhile, we use the Lux 5 µm Cellulose-4 chiral column to separate 7-hydroxyflumequine which is the main metabolite of flumequine. There is no report on the chiral analytical methods of flumequine and its metabolites enantiomers in water and sediment. To the best of our knowledge, the current report is the first time to simultaneous present the enantioselective analysis of flumequine and its metabolite in water and sediment samples using chiral HPLC-Q-TOF/MS. This method which will be potentially beneficial for research in this area will be sensitive, selective, not so much time-consuming and easily applicable to analyze water and sediment samples.

## Experimental

### Chemical reagents and apparatus

Racemic flumequine standard (purity at 99.9%) and S-(−) and R-(+)-flumequine enantiomers (purity >90.0%) were obtained from CNW Technologies. Racemic 7-hydroxyflumequine were prepared by Peking University Health Science Center. Acetonitrile, methanol and n-hexane (HPLC grade) were purchased from Sigma Aldrich (USA). The HPLC water was prepared through a Milli-Q system (Millipore, MA, USA). All other chemicals and reagents were of analytical grade supplied by Guoyao Chemical Co. (Shanghai, China). 0.22 µm Filter Unit was from Bonna-Agela Technologies Co.,Ltd, (Beijing, China). Cleanert PEP Solid phase extraction cartridges (500 mg/6 mL) were purchased from Bonna-Agela Technologies. High-speed refrigerated centrifuge (CR22G) was purchased from Hitachi Led. (Tokyo, Japan). A 5600 Accurate-mass tandem quadrupole-time-of-flight (Q-TOF) mass spectrometer (SCIEX, Co., Ltd.) was used to quantify flumequine enantiomers. JASCO 2000 HPLC (Jasco Co., Tokyo, Japan) equip with circular dichroism detector (CD-2095).

### Preparation of working standards

Standard solutions of racemic flumequine, racemic 7-hydroxyflumequine, S-(−) and R-(+)-flumequine enantiomers were prepared in pure ACN, obtaining a final concentration of 200.0 µg/L. All solutions were protected against light and stored in the dark at 4 °C.

### Validation of method

The linear range, limit of detection (LOD), limit of quantification (LOQ), specificity, accuracy and precision were determined to validate the performance of the method.

The linearity of the method was evaluated based on the peak areas of the matrix-matched standard solutions in triplicate at eight concentration levels, ranging from 1.0 to 200.0 µg/L. The LOD was the concentration that produced a signal-to-noise (S/N) ratio of 3, whereas the LOQ was based on a S/N ratio of 10^41^.

The recovery assay was determined by spiking the flumequine and 7-hydroxyflumequine enantiomers analytes into sediment samples at 10, 50, 100 µg/L and 5, 10, 20 µg/L for water samples. Six spiked samples on each level were extracted as described above and the recovery of each one was calculated. The concentration of target enantiomers were then determined using the external standard calibration to obtain the recovery and accuracy. The relative standard deviations (RSDs) of the measured values were calculated to evaluate the precision of the method. The repeatability was calculated using the data obtained from the recovery. The precision was calculated using the same data obtained for the recovery and repeatability.

When using LC-MS/MS to measure complex samples, the matrix usually has an enhancing or inhibiting effect on the ionization of the analyte, which is the matrix effect. The matrix effect affects the sensitivity and repeatability of the instrument, which is an important factor influencing the reliability and accuracy of the instrument.

Therefore, the matrix effects (MEs) can be evaluate by using the formula (the slope of the curve obtained from the matrix matched standard solution/the slope of the curve made of the non-matrix standard solution − 1) × 100%, with a matrix-induced effect when the value is negative and matrix enhancement occurring when the value is positive.

### Samples collection

Sediment samples were obtained from the pool in Tianjin. Using the bottom sampler to collect sediment samples at the bottom of the pool. All samples were refrigerated storage at 4 °C and return to the laboratory. These samples did not contain the target analytes. After the natural drying process, the sediment samples were homogenized into powder and were passed through mesh sieve and stored in the refrigerator at −20 °C until analysis.

### Sample preparation

#### Sediment

Dry sediment samples (2.00 ± 0.01 g) were weighed into 50 mL centrifuge tube, and then 10 mL ACN and EDTA-Mcllvaine buffer solution (40:60, v/v) were added to the tube. Subsequently, the mixtures were homogenized for 1–2 min and were extracted by an ultrasonic oscillator for 10 min. The mixtures were further centrifuged at 8000 rpm for 5 min and the above steps are repeated three times then the extraction solution was transferred into 150 mL round-bottom flask for drying using a rotary evaporator (45 °C). After that, diluted with water to 30 mL, pour into 50 mL centrifuge tube until serve.

Cleanup with Cleanert PEP (Polar Enhanced Polymer) cartridge. For this analysis, 30 mL extraction solution was slowly passed through Cleanert PEP cartridges, the flow rate is about no more than 2 mL/min, which was previously activated by sequential flushing with 6 mL MeOH and 6 mL purified water. After the sample was loaded, the SPE cartridge was washed with 6 mL 0.2%Acid Aqueous Solution, The retained analytes were eluted with 6 mL MeOH. The organic solvent was dried by pure nitrogen, then the resultant residue was re-dissolved in 1 mL MeOH and filtered through a 0.22 µm filter for HPLC-Q-TOF/MS analysis and quantitation.

#### Water

50 mL water samples were cleaned-up with Cleanert PEP cartridge, the process was as described above. Flumequine and 7-hydroxyflumequine have the same process of extraction and cleanup.

### Q-TOF-MS analysis

#### Flumequine analysis

Chromatographic separations were carried out on Lux 5 µm Cellulose-2 (250 mm × 4.6 mm i.d. × 5 µm, Phenomenex, USA) column. The mobile phase consisted of 0.2% acetic acid in water as solvent A and acetonitrile as solvent B. The gradient elution program was as follows: 0–20 min, A:B(45:55,V/V); 20–24 min, A:B(5:95, V/V); 24–25 min, A:B(45:55, V/V); The total run time was 30 min at a flow of 1 mL/min. The separation is isocratic, using a wash step with a high acetonitrile concentration to clean the column. The column temperature was maintained at 30 °C. The injected volume of the test sample was set at 1 µL. Q-TOF detection equipped with an electrospray ionization source (ESI) was performed in positive ion mode, nebulizer pressure, 55 psi; collision energy, 10 V; capillary voltage, 5.5 kV; ion temperature, 600 °C; the mass range of m/z 100–700.

#### hydroxyflumequine analysis

The spatial configuration of flumequine and 7-hydroxyflumequine is different, so different chiral stationary phase are needed to separated them. We choose normal-phase chromatography elution mode and the Lux 5 µm Cellulose-4 (250 mm × 4.6 mm i.d. × 5 µm, Phenomenex, USA) column to separate 7-hydroxyflumequine. The mobile phase consisted of 0.3% TFA in ethyl alcohol as solvent A and n-hexane as solvent B. The total run time was 20 min at a flow of 1 mL/min. The column temperature was maintained at 30 °C. The injected volume of the test sample was set at 1 µL. Q-TOF detection equipped with an electrospray ionization source (ESI) was performed in positive ion mode, nebulizer pressure, 55 psi; collision energy, 10 V; capillary voltage, 5.5 kV; ion temperature, 600 °C; The mass range of m/z 100–700.

## Results and Discussion

### Separation condition optimization and elution order

In the preliminary experiments, the enantioseparation of flumequine on two cellulose-based columns (Lux 5 µm Cellulose-2 and Lux 5 µm Cellulose-3, 250 mm × 4.6 mm i.d. × 5 µm particle size) was tested. Cellulose-2 and Cellulose-3 both are derivatives of polysaccharides chiral column. Cellulose-3 column has one cellulose benzoate-based chiral selector. However, it is an effective way to increase the hydrogen bonding between solute and stationary phases to introduce into amino in chiral stationary phases for Cellulose-2 column. Hence, better chromatographic separation of flumequine enantiomers can be achieved when Lux 5 µm Cellulose-2 is used. So, optimization of chromatographic conditions below was subsequently conducted using a Lux 5 µm Cellulose-2 column. The chromatographic separation behavior was to some extent affected by changing the amount or type of organic and acidic modifiers in the mobile phase. When using acetonitrile as the organic mobile phase B with the mobile phase ratios of A:B varied from 10:90 to 50:50, the resolutions (Rs) for flumequine enantiomers were range from 0.63 to 2.01. In the ratio of 50:50, an acceptable Rs value (2.01) result indicated that the flumequine enantiomers were completely separated with the mobile phase was acetonitrile and 0.2% acid aqueous (50:50).

**Figure 3:**
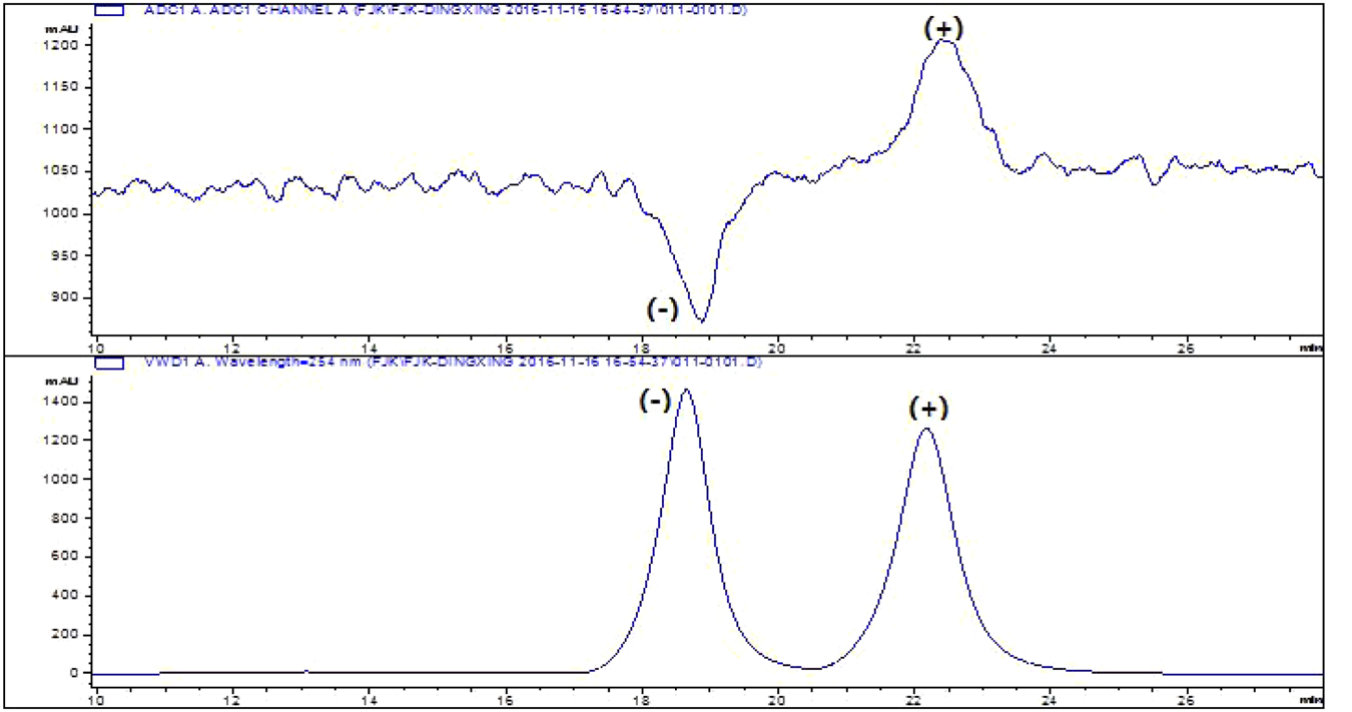
The CD and UV chromatogram of flumequine on Lux Cellulose-2.

By coupling the chiral LC to the optical rotation detection and UV detection sequentially using the same stationary and mobile phases, both (−)enantiomer forms were eluted out earlier than the (+) forms for chiral flumequine (Fig. 3).

**Figure 4:**
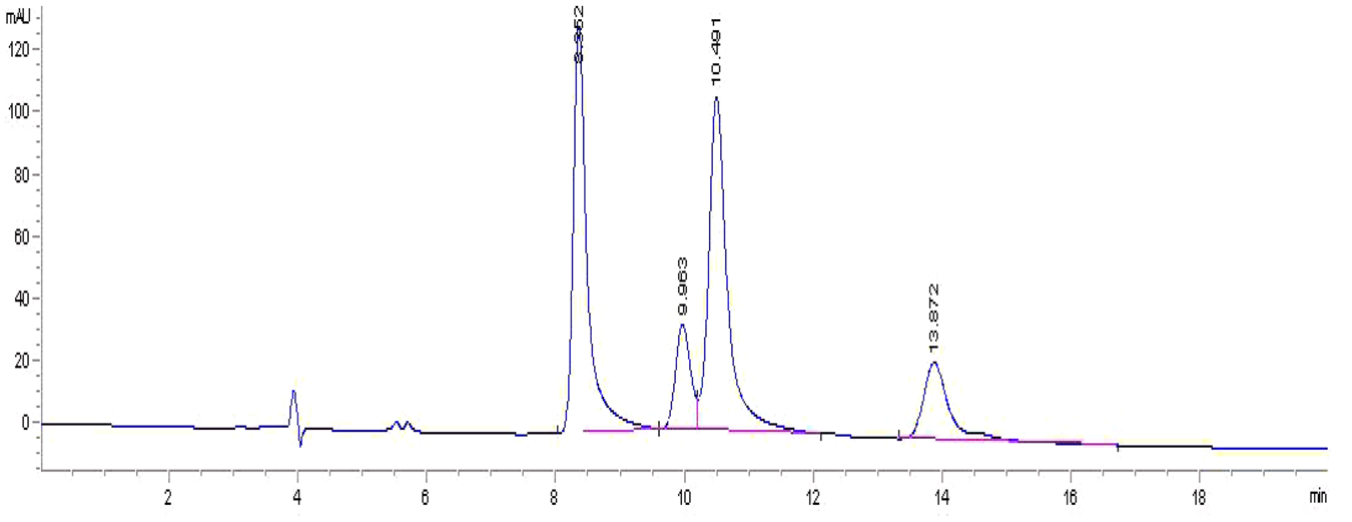
The separation of 7-hydroxyflumequine was performed on Lux Cellulose-4.

The enantioseparation of 7-hydroxyflumequine on the Lux 5 µm Cellulose-4 column. The mobile phase ratio of A:B was varied from 40:60 to 10:90. It was found that each condition did not have a significant effect on the enantioselective separation efficiency. However, the better chromatographic separation of 7-hydroxyflumequine enantiomers can be achieved when TFA in the mobile phase. Therefore, the ethyl alcohol solution containing amount of TFA (0.3%) was chosen for the solvent A. In addition, the optimization of mobile phase ratio were tested, by comparison, the best chromatographic enantioseparation for the four enantiomers of the 7-hydroxyflumequine in the ratio of 70:30 (Fig. 4).

### Mass spectrometric analysis of flumequine

**Figure 5:**
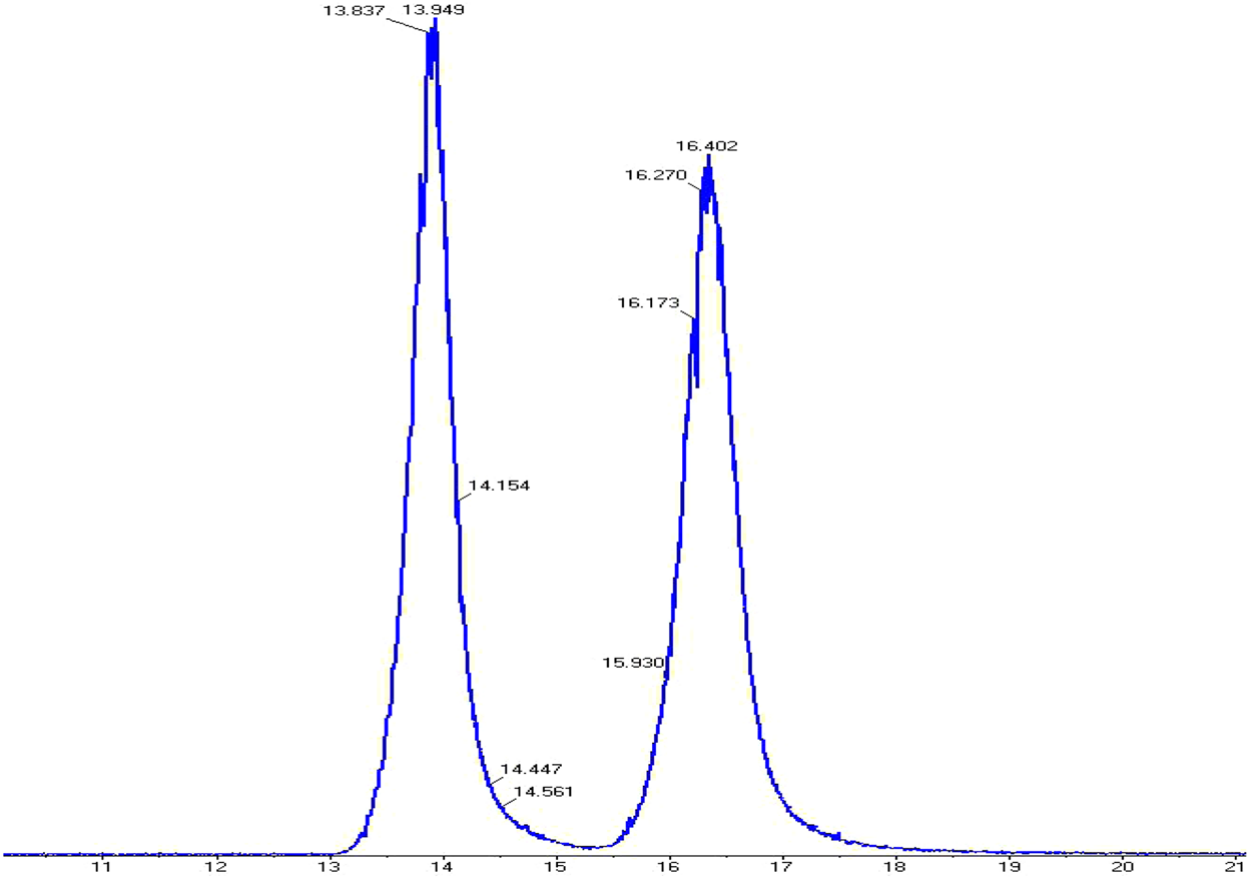
The separation was performed on Lux Cellulose-2.

**Figure 6:**
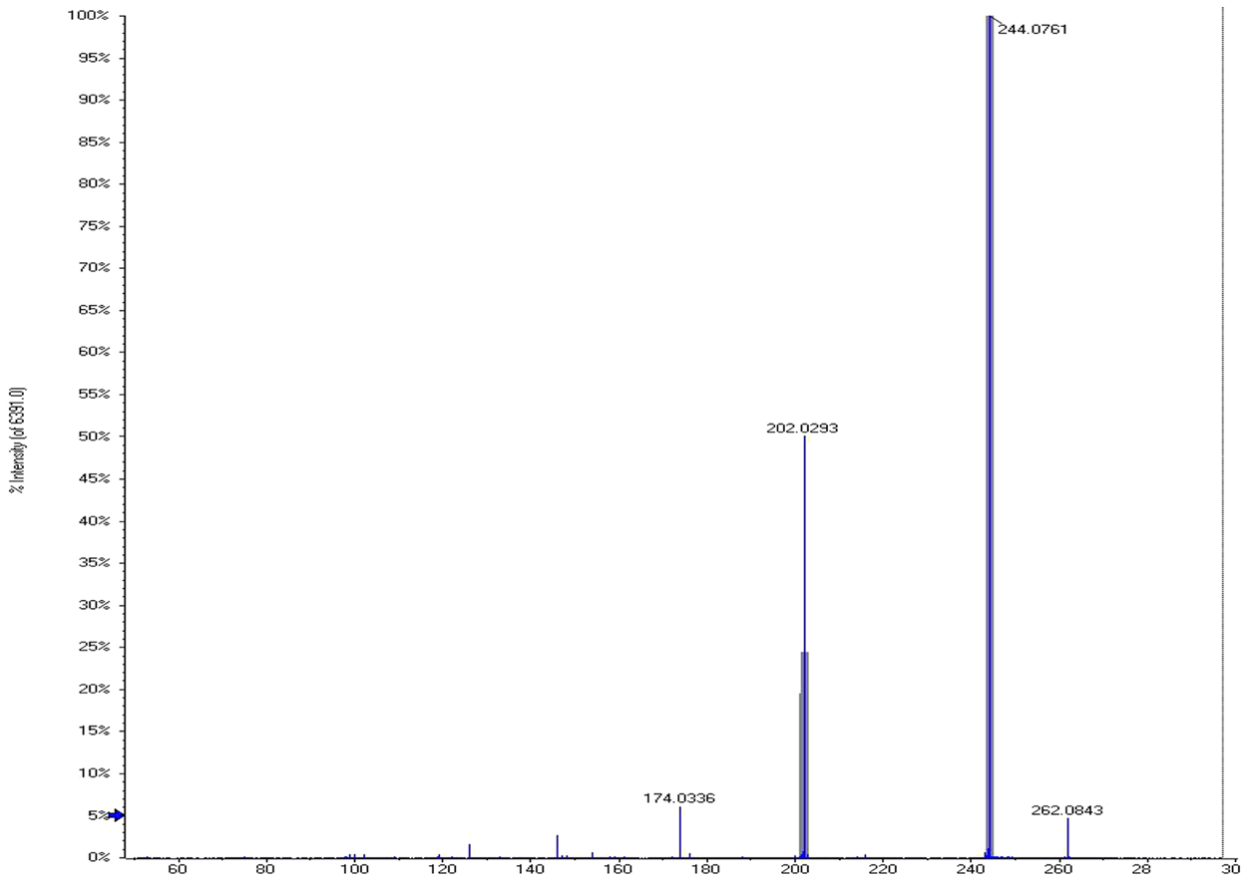
MS/MS spectra of flumequine (Abscissa: counts vs mass-to-charge m/z; Ordinate: Intensity).

The MS/MS analysis of protonated flumequine was carried out by HPLC/QTOF-MS. Mobile phase A is 0.2% acetic acid solution and mobile phase B is ACN. The flow rate was set at 1 mL/min and the column temperature was set at 30 °C. Elution of the two enantiomers was observed within 13.9 min (the first effluent fraction, *S*-(−)-Flumequine) and 16.4 min (the second effluent fraction, *R*-(+)-Flumequine) (Fig. 5). The margin of error between the measured and calculated masses ranged from −0.2 to 0mDa, indicating good accuracy. The protonated molecular ions ([M + H]^+^) and the fragment ions of [M + H]^+^ were all measured (Fig. 6). The most possible elemental compositions and the product ions were listed in Table 1.

**Table 1:** Elemental composition, measured and calculated masses, and mass errors of protonated flumequine and its fragment ions.

Elemental compositon ([M + H]^+^)	Measured M_W_ (Da)	Theoretical M_W_ (Da)	Error (mDa)	Error (ppm)	Collision energy /eV
C_14_H_12_NO_3_F	262.0873	262.0874	−0.1	−0.4	92
C_10_H_5_NOF	174.0333	174.0349	−1.6	−9.5	18
C_11_H_5_NO_2_F	202.0293	202.0298	−0.5	−2.6	61
C_14_H_11_NO_2_F	244.0761	244.0768	−0.7	−2.9	55

### Sample preparation optimization

Sample preparation is the crucial step in environmental analysis. It is highly influenced by the physical and chemical properties of analytes studied. The main goal is to concentrate analytes in sample, to remove interferences from matrix and to prepare analyte in suitable form for subsequent chromatographic analysis^42^.

Acetonitrile/ethyl acetate (1: 1, V/V) and acetonitrile/PBS buffer solution (1: 1, V/V) as extracting agent to examine the efficiency. When the acetonitrile/PBS buffer solution was used as the extracting agent, the extraction recoveries for the flumequine enantiomers range from 13.4~17.7%, and for the 7-hydroxyflumequine range from 11.2~13.5%. However the extraction efficiency of acetonitrile/ethyl acetate for the two drugs were not good.

When using ACN/EDTA-Mcllvaine solution as extraction solvent, the extraction efficiency is satisfied. And then, optimize the ratio of ACN and EDTA-Mcllvaine solution, select the ratio of ACN and EDTA-Mcllvaine solution were 10:0, 9:1, 8:2, 7:3, 6:4, 5:5. It was found that when the ratio was 6:4 the recoveries for the two drugs were the highest. (Table 2)

**Table 2:** The average recoveries and RSD% of flumequine and 7-hydroxyflumequine at different extraction solvent ratios. (n = 3) .

Extraction Solvent	Flumequine			7-hydroxyflumequine		
	Recovery(%)	RSD(%)	CV%	Recovery(%)	RSD(%)	CV%
ACN/EDTA-Mcllvaine (10:0, V/V)	39.70	1.73	4.37	40.20	1.60	3.98
ACN/EDTA-Mcllvaine (9:1, V/V)	37.77	5.23	13.85	42.85	6.70	15.64
ACN/EDTA-Mcllvaine (8:2, V/V)	47.27	6.35	13.44	35.65	3.21	9.00
ACN/EDTA-Mcllvaine (7:3, V/V)	60.04	2.25	3.75	67.7	4.21	6.22
ACN/EDTA-Mcllvaine (6:4, V/V)	77.93	1.85	2.37	76.69	3.27	4.26
ACN/EDTA-Mcllvaine (5:5, V/V)	61.30	0.40	0.65	64.7	1.17	1.81

### Solid phase extraction cartridges optimization

In order to select appropriate capacity of the SPE cartridges, after rotary evaporator drying and diluted with water, then using the above optimized preparation method to process the samples. The cartridges recoveries experiments were carried out using three solid phase extraction cartridges, Sep-Pak C18 (500 mg, 6 mL), Poly-sery HLB (60 mg, 3 mL), Cleanert PEP (150 mg, 6 mL), respectively. Through the further enrichment of the C18 cartridges filtrate, it was found that there was a large amount of the antibiotic residue in the filtrate, which leads to loss some analytes and low recovery rates, therefore C18 cartridges was unable to absorb flumequine and 7-hydroxyflumequine in the extraction solvent. The recoveries of flumequine enantiomers through an HLB cartridges range from 17.8~22.2%, and of the 7-hydroxyflumequine range from 13.4~14.3%. Here, PEP cartridge was chosen as the absorbent for the flumequine enantiomers and 7-hydroxyflumequine infusion samples because of its satisfied recovery range from 77~81.8%, 70.1~78.69%, respectively.

Classical C18 sorbent is the most commonly used SPE column which chemistries was bonded silica. Silica based sorbents are not suitable for the extraction of quinolone antibiotics because of it was effective only for non-polar compounds. Cleanert PEP (Polar Enhanced Polymer) are functionalized polystyrene/divinylbenzene extraction cartridges which show the most robust recovery ratio and reproducibility for both polar and non-polar compounds, and they are allowing working in wide range of pH (from pH 1 to 14). However, comparing to the HLB cartridges, PEP cartridges were much more efficient, yielding high recoveries for flumequine enantiomers and 7-hydroxyflumequine. Therefore, this kind of cartridges can also be used for the extraction of other fluoroquinolones antibiotics.

### Method validation

#### Recoveries and precision

The recoveries of method were evaluated by spiking the blank samples at three different concentration levels of the flumequine and 7-hydroxyflumequine (10, 50, 100 µg/L for sediment samples and 5, 10, 20 µg/L for water samples). Intra - day precision were obtained by measuring 6 replicates of different matrices at 3 spiked levels within one day. Inter - day precision were obtained for 5 consecutive days. The results of the average recovery, standard deviation and relative standard deviation of the studied flumequine and 7-hydroxyflumequine are summarized in Table 3. The method presented satisfactory mean recoveries values from 71.7 ± 12.5% to 84.6 ± 5.6% for both flumequine enantiomers in different matrix. The intra - day precision were range from 5.1~12.2%, and the inter - day precision were range from 4.5~16.4%. The results of precision for 7-hydroxyflumequine were also appropriate, the mean recoveries values from 69.9 ± 13.1% to 83.9 ± 3.5% in different matrix. The intra - day precision and inter - day precision were range from 3.5~13.1%, 3.0~7.6%, respectively.

**Table 3:** Spiked average recoveries and relative standard deviations(RSDs) of flumequine and 7-hydroxyflumequine. (n = 6).

Sample	Spiked (µg/L)	Flumequine				7-hydroxyflumequine			
		Intra - day		Inter - day		Intra - day		Inter - day	
		Recovery (%)	RSD	Recovery (%)	RSD	Recovery (%)	RSD	Recovery (%)	RSD
Water	5	79.7	12.2	82.6	5.3	76.3	4.7	73.1	6.2
	10	84.6	6.7	73.1	4.5	73.3	5.1	77.5	4.9
	20	83.8	5.1	84.6	5.6	83.9	3.5	77.7	5.8
Sediment	10	73.6	6.3	71.7	12.5	69.9	13.1	70.3	3.0
	50	74.3	12.2	73.2	5.1	76.2	6.2	73.4	7.6
	100	77.5	11.9	76.0	16.4	71.8	7.4	72.3	7.4

#### Matrix effect

This manuscript evaluated the matrix effects of each enantiomer of flumequine and racemic 7-hydroxyflumequine in sediment samples (Table 4). It was shown that there were matrix-induced effect for the enantiomers of flumequine and 7-hydroxyflumequine. The difference in matrix effects of two enantiomers of flumequine are not significant. When all of these problems are considered together, we adopted the method of preparing matrix matched standard solution which can eliminate the effect of the matrix and can meet the requirement of residual detection completely.

**Table 4:** Evaluation of matrix effects of flumequine and 7-hydroxyflumequine in sediment.

	Calibration curve without matrix	R^2^	Calibration curve with matrix	R^2^	Matrix effect(%)
*S*-(−)-flumequine	Y = 318748x − 281641	0.9944	Y = 152208x − 211323	0.9915	−52.2
*R*-( + )-flumequine	Y = 344863x − 318334	0.9919	Y = 187164x − 177560	0.9927	−45.7
7-hydroxyflumequine	Y = 55563x − 50519	0.9936	Y = 39069x − 39706	0.9822	−29.7

#### Linearity, LOD and LOQ

We obtained a very good linearity within the concentration range of 1.0 to 200.0 µg/L(1.0, 2.0, 5.0, 10, 20, 50, 100, 200) for each enantiomer of flumequine and 7-hydroxyflumequine prepared in the matrix-matched solvent in water and sediment samples were satisfactory. The calibration curves and correlation coefficients for flumequine enantiomers and 7-hydroxyflumequine in different matrix were showed in Table 5.

**Table 5:** The linear regression equations, regression coefficients (R^2^) and limits of detection (LODs) for enantiomers of flumequine and 7-hydroxyflumequine in different samples by HPLC-Q-TOF/MS determination.

Enantiomers	Samples	Regression equation	R^2^	LOD (µg/kg or µg/L)
*S*-(−)-Flumequine	Water	Y = 23910x − 1181.7	0.9954	2.5
	Sediment	Y = 152208x − 211323	0.9915	5.0
*R*-(+)-Flumequine	Water	Y = 52291x − 48709	0.9927	2.5
	Sediment	Y = 187164x − 177560	0.9927	5.0
7-hydroxyflumequine	Water	Y = 91664x− 118565	0.9988	3.2
	Sediment	Y = 39069x − 39706	0.9822	7.0

In water samples, the values obtained for the LOD were 2.5 µg/L for S-(−)-Flumequine and R-(+)-Flumequine, respectively, and for LOQ they were 8.0 µg/L for two enantiomers. In sediment samples, the LOD and LOQ were 5.0 µg/kg and 15.0 µg/kg for both the enantiomers. The LOD for 7-hydroxyflumequine was estimated as 3.2 µg/L in water samples, and as 7.0 µg/kg in sediment samples. The LOQ were 10 µg/L and 20 µg/kg in water and sediment samples respectively.

### Application

#### Application to the real sediment samples

**Figure 7:**
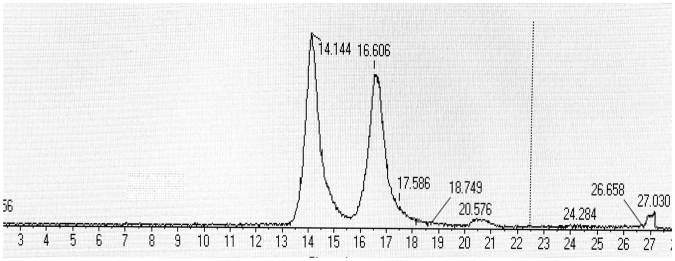
The HPLC-Q-TOF/MS chromatograms of flumequine obtained from real sediment samples.

A total of three different sediment samples were obtained from different pools of Xiqing District of Tianjin. The samples were extracted and cleaned-up according to Sections 2.4 and 2.5 followed by simultaneous and enantioselective determination of flumequine. The HPLC-Q-TOF/MS chromatograms obtained from the sediment samples at different times after application of flumequine enantiomers are shown in Fig. 7. The resolution between (+)enantiomer and (−)enantiomer was greater than 2.0 under all separation conditions tested, demonstrating sufficient robustness. The results suggest that the chiral method for the separation and determination of flumequine enantiomers, which are characterized by high sensitivity and specificity, rapidity, and possibility of performing the simultaneous analysis of water and sediment samples, become more and more important.

## Conclusions

In the present work, chiral separation methods were developed for the determination of flumequine and 7-hydroxyflumequine enantiomers, which were validated for the simultaneous and quantitative determination of the enantiomers of flumequine and its main metabolite 7-hydroxyflumequine enantiomers in water and sediment. The method showed good performance regarding the linearity and instrumental repeat ability.

Meanwhile, effects of the sample preparation and solid phase extraction cartridges were optimized, the recoveries were found to be good with Cleanert PEP. The effect extraction solvent of the sample was studied and found that the ACN and EDTA-Mcllvaine solution was more suitable for extraction of all the test compounds. Experimental results showed that the determination method has high sensitivity and low detection. Moreover, the method developed can be applied to Simultaneous analysis of flumequine antibiotic and its metabolite in water and sediment matrices.
